# Association between Immunoglobulin GM and KM Genotypes and Placental Malaria in HIV-1 Negative and Positive Women in Western Kenya

**DOI:** 10.1371/journal.pone.0053948

**Published:** 2013-01-11

**Authors:** Nnaemeka C. Iriemenam, Janardan P. Pandey, John Williamson, Anna J. Blackstock, Ajay Yesupriya, Aryan M. Namboodiri, Keith M. Rocca, Anna Maria van Eijk, John Ayisi, Juliana Oteino, Renu B. Lal, Feiko O. ter Kuile, Richard Steketee, Bernard Nahlen, Laurence Slutsker, Ya Ping Shi

**Affiliations:** 1 Division of Parasitic Diseases and Malaria, Center for Global Health, Centers for Disease Control and Prevention, Atlanta, Georgia, United States of America; 2 Department of Microbiology and Immunology, Medical University of South Carolina, Charleston, South Carolina, United States of America; 3 Atlanta Research and Education Foundation/VA Medical Center, Decatur, Georgia, United States of America; 4 National Office of Public Health Genomics, Coordinating Center for Health Promotion, Centers for Disease Control and Prevention, Atlanta, Georgia, United States of America; 5 Centre for Vector Biology and Control Research, Kenyan Medical Research Institute, Kisumu, Kenya; 6 New Nyanza Provincial General Hospital, Ministry of Health, Kisumu, Kenya; 7 Division of AIDS, STD, TB Laboratory Research, National Center for HIV, STD, TB Prevention, Centers for Disease Control and Prevention, Atlanta, Georgia, United States of America; 8 Child and Reproductive Health Group, Liverpool School of Tropical Medicine, Liverpool, United Kingdom; Instituto de Ciências Biomédicas/Universidade de São Paulo - USP, Brazil

## Abstract

Immunoglobulin (Ig) GM and KM allotypes, genetic markers of γ and κ chains, are associated with humoral immune responsiveness. Previous studies have shown the relationships between GM6-carrying haplotypes and susceptibility to malaria infection in children and adults; however, the role of the genetic markers in placental malaria (PM) infection and PM with HIV co-infection during pregnancy has not been investigated. We examined the relationship between the gene polymorphisms of Ig GM6 and KM allotypes and the risk of PM infection in pregnant women with known HIV status. DNA samples from 728 pregnant women were genotyped for GM6 and KM alleles using polymerase chain reaction-restriction fragment length polymorphism method. Individual GM6 and KM genotypes and the combined GM6 and KM genotypes were assessed in relation to PM in HIV-1 negative and positive women, respectively. There was no significant effect of individual GM6 and KM genotypes on the risk of PM infection in HIV-1 negative and positive women. However, the combination of homozygosity for GM6(+) and KM3 was associated with decreased risk of PM (adjusted OR, 0.25; 95% CI, 0.08–0.8; *P* = 0.019) in HIV-1 negative women while in HIV-1 positive women the combination of GM6(+/−) with either KM1-3 or KM1 was associated with increased risk of PM infection (adjusted OR, 2.10; 95% CI, 1.18–3.73; *P* = 0.011). Hardy-Weinberg Equilibrium (HWE) tests further showed an overall significant positive F(is) (indication of deficit in heterozygotes) for GM6 while there was no deviation for KM genotype frequency from HWE in the same population. These findings suggest that the combination of homozygous GM6(+) and KM3 may protect against PM in HIV-1 negative women while the HIV-1 positive women with heterozygous GM6(+/−) combined with KM1-3 or KM1 may be more susceptible to PM infection. The deficit in heterozygotes for GM6 further suggests that GM6 could be under selection likely by malaria infection.

## Introduction

Malaria is a major global public health problem and affects sub-Saharan Africa predominantly [1]. Annually, about 125 million pregnant women living in malaria-endemic areas, with 32 million in sub-Saharan Africa, are at risk of malaria [2], and nearly 10,000 of these women and 200,000 infants die due to the direct or indirect consequences of maternal *Plasmodium falciparum* infection [2], [3]. Inhabitants of malaria-endemic regions usually develop protective immunity. However, because this protection is decreased during pregnancy, pregnant women are at higher risk of malaria infection than their age-matched non-pregnant women [4], with malaria parasites sequestering in the placenta due to the selection of pregnancy-associated *P. falciparum* erythrocyte membrane protein-1 (*Pf*EMP-1) variant surface antigen (VSA) [5]. Placental malaria (PM) is often associated with maternal illness and fetal loss in low transmission areas; in high transmission areas, most mothers are asymptomatic but suffer from maternal anemia, preterm labor, and low-birth weight infants contributing to increased risk of neonatal and infant morbidity and mortality [3], [6], [7]. Malaria and human immunodeficiency virus (HIV) infection often overlap in women of reproductive age in many sub-Saharan African countries, emphasizing the public health importance of this co-infection. Co-infection with HIV in pregnant women increases the risk of malaria infection, high parasite density, maternal anemia, low-birth weight infants and congenital malaria infection [8].

Although the immunological mechanisms responsible for eliminating parasites from the placenta remain unclear, humoral immune responses have been implicated. Unlike multigravidae, primigravid women are more susceptible to PM and are less likely to have the antibodies that inhibit the binding of infected red blood cells to chondroitin sulfate A (CSA) in the placenta. The previous studies on parity-dependent antibody responses further suggest the existence of pregnancy-associated VSA expressed on infected red blood cells [9], [10], [11]. In addition, naturally acquired antibody responses to other blood stage antigens such as the C terminus of merozoite surface protein 1_19_ (MSP-1_19_) have been shown to be associated with reduced PM infection [11]. However, HIV infection selectively alters humoral immune functions [12], diminishes the development of parity-dependent antibody responses to pregnancy-associated VSA and impairs humoral responses to other malaria antigens in pregnant women [13], [14]. Furthermore, the effect of gene polymorphism in Fc receptors for antibody IgG (FcγRs) has been investigated in malaria during pregnancy with or without HIV co-infection [15]. FcγRs are expressed on monocytes, macrophages, B-cells, and other leukocytes and by binding to the antibody Fc-portion, they provide an important link between the humoral and cellular arms of the immune system for effector function and for immune modulation [16]. Our previous report showed that the genotype of FcγRIIa-H/H131 was associated with enhanced susceptibility to PM in HIV-positive women while the same FcγRIIa genotype was found to be less important for PM in HIV-negative women [15]. These results indicate that the particular genotype of FcγRIIa and possible polymorphisms in the Fc portion of IgG may epistatically interact, contributing to the disease-specific effector responses mediated by IgG molecules [17].

Polymorphisms in the Fc portion of IgG called Ig GM and KM allotypes are genetic markers of γ and κ chains, inherited as autosomal codominant genes according to Mendelian laws [18]. GM allotypes are encoded by three very closely linked cistrons on chromosome 14. They are localized on the constant region of γ1, γ2 and γ3 heavy chains, and there are currently 18 testable GM specificities [19]. Linkage disequilibrium in the GM system is almost absolute, and the determinants are transmitted as a group called GM haplotypes. KM determinants are inherited via three alleles on chromosome 2–KM1, KM1,2 and KM3. The clinical importance of Ig GM and KM allotypes has been evaluated for a number of other infectious diseases such as infections with hepatitis C virus or *Haemophilus influenzae* type b bacteria. These studies showed strong associations between specific Ig GM and KM polymorphisms and susceptibility to and outcome of infections [19], [20]. However, their roles in PM infection and PM with HIV co-infection have not been investigated. A previous study found an inverse relationship between the carriage of the GM5,6,13,14 (expressed on IgG3);1,17(expressed on IgG1) phenotype and uncomplicated malaria in children [21], and results from another study indicated that the critical element in differences in susceptibility to malaria infection seen between two sympatric tribes in eastern Sudan might be GM6-carrying haplotypes [22]. In addition, the geographic distribution of GM6-related haplotypes coincides with the region of high falciparum malaria, sickle-cell allele and G6PD deficiency prevalence [23], [24]. GM6 is also confined in sub-Saharan Africa and rarely found in other continents, with the exception of African-Americans in North America [24].

Given the relevance of GM6 in malaria infection in non-pregnant populations [21], [22], [23] and the possibility that Ig GM and KM allotypes may contribute to the risk of PM infection and PM with HIV co-infection by epistatic interaction with FcγR [15], [17], the aim of this study was to investigate the association between gene polymorphisms of Ig GM6, KM allotypes and PM infection in Kenyan pregnant women with known HIV status. In addition, we further evaluated possible selection by malaria on Ig GM6 and KM in the study population.

## Patients and Methods

### Ethics Statement

This study was approved by the Ethical Review Committee of the Kenya Medical Research Institute, Nairobi, Kenya and the Institutional Review Board of the Centers for Disease Control and Prevention, Atlanta, Georgia, USA. Informed written consent was obtained from all study participants.

### Study Participants and Data Collection

This study was integrated into an observational cohort study (VT project) investigating the effect of PM on perinatal mother-to-child transmission of HIV-1 in western Kenya. Details of the study design, population and clinical procedures have been published elsewhere [15], [25]. Briefly, pregnant women attending the antenatal clinic at New Nyanza Provincial General Hospital in Kisumu from 1996–2001 were enrolled. Inhabitants of this area are predominantly of the Luo ethnic tribe. At enrollment, a questionnaire was administered to extract information on reproductive history, socio-demographics, behavior, and clinical status. Blood samples were taken from mothers for HIV-antibody testing, as well as malaria blood smears and hemoglobin level. At delivery, blood samples were collected from the periphery, placenta and cord to determine placental parasitemia, hemoglobin level and viral load. One month postpartum, additional blood samples were obtained from the mothers for CD4 cell counts, hemoglobin and malaria diagnosis. The maternal samples collected at delivery were used for the current genetic study. The VT cohort study originally enrolled 269 HIV-negative and 829 HIV-positive pregnant women. The difference in the number of women in the two groups was due to the fact that enrollment priority was given to HIV-positive women. For the HIV-negative women, enrollment priority was given to the women with PM. For the present genetic study, only those women who had maternal DNA samples and clinical data with no missing information on antimalarial drug treatment during pregnancy were included. Four groups of pregnant women in the current study are: (a) 132 HIV-1 negative women with PM, (b) 107 HIV-1 negative women without PM, (c) 119 HIV-1 positive women with PM, and (d) 370 HIV-1 positive women without PM.

### Laboratory Procedures

#### Malaria and anemia diagnosis

PM was assessed on blood samples obtained from a shallow incision on the maternal side of the placenta. Thick smears were made from the maternal and placental blood and examined by microscopy. Parasite densities were calculated based on 8,000 leukocytes/µL blood having counted 300 leukocytes. Placental smear readings were also categorized on the basis of malarial pigment in placental intervillous macrophages [26]. Peripheral blood hemoglobin concentrations were measured using HemoCue system (HemoCue).

#### HIV-1 serological diagnosis, CD4 count and viral load

Maternal HIV-status was determined by a primary Serotrip HIV-1/2 (Saliva Diagnostic Systems) and a confirmatory Capillus HIV-1/2 test (Cambridge Diagnostics). Maternal CD4 counts were determined by the use of commercial monoclonal antibodies and standard fluorescent-activated cell sorting analysis of whole blood (FACScan, Becton Dickinson). Maternal HIV-1 load was measured by use of the Roche Amplicor HIV-1 monitor (test version 1.0; Roche Diagnostics).

#### GM6 and KM genotyping

**Figure 1:**
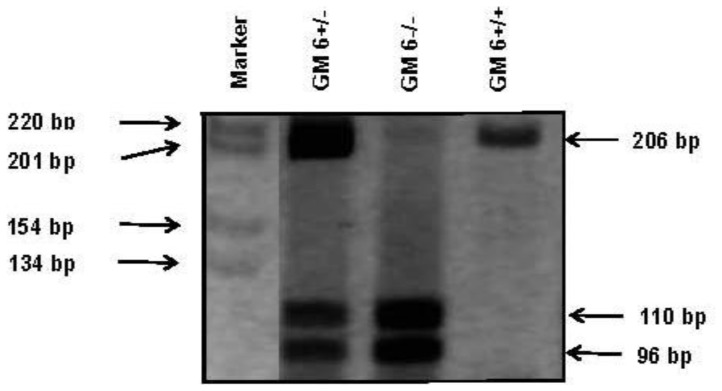
Restriction fragment length polymorphism analysis for GM6 markers.

Genomic DNA was purified from frozen blood pellets by micro-centrifugation using a commercial DNA purification mini-kit QIAmp® (Qiagen). Since there are no published methods for molecular genotyping of GM6, a polymerase chain reaction-restriction fragment length polymorphism (PCR-RFLP) method was developed for this investigation based on the amino acid sequence information provided by Dard *et al*. [27]. According to these authors, GM6 is determined by a glutamine (CAG) to glutamic acid (GAG) substitution at position 419 (CH3 region) of IgG3. The position of the GM6 C/G SNP has a unique cutting site for the restriction enzyme Fnu4HI. Two sites were chosen, where the IgG3 gene differed from the genes for other IgG subclasses, for use as forward and reverse primers. Briefly, the DNA segment encoding the CH3 region of human γ3 chain determinant GM6 gene was amplified by PCR using the following primers: Forward primer: 5'-GCGGGCAGCCGGAGAACAACTACAAC-3' and Reverse primer: 5'-GCTTGCCGGCTATCGCACTC-3'. After digestion with Fnu4HI and the fragments separated on 8% polyacrylamide gel, the following products corresponding to the three genotypes were detected: GM6[+] (206-bp fragment), GM6[+/−] (206-bp, 110-bp and 96-bp fragment) and GM6[−] (110-bp and 96-bp fragment) (Figure 1). For quality control of this RFLP method for GM6, we randomly sequenced 133 samples; of these, 118 matched the PCR-RFLP results. With the remaining 15 samples, the results were inconclusive because the quality of sequencing was not acceptable. To our knowledge, GM6 is found exclusively in the populations of African descent. Therefore, in another measure of quality control for the GM6 RFLP method, we genotyped 114 HIV-infected Caucasian subjects for GM6; none was positive for this determinant. κ chain determinants KM1 and KM3 were characterized by the PCR-RFLP technique published by Moxley and Gibbs using the following primers: 5'-TAGGGGGAAGCTAGGAAGAAA-3' and 5'-AAAAAGGGTCAGAGGCCAAA-3' for PCR [28]. After digestion of the amplified product (538-bp) with the restriction enzyme Acc1, the following products corresponding to the three genotypes were detected: KM1 (538-bp fragment), KM3 (390-bp and 148-bp fragment) and KM1-3 (538-bp, 390-bp and 148-bp fragment).

### Definitions

#### Clinical definitions

Placental parasitemia was defined as *P. falciparum* asexual form detected on a thick film. Peripheral parasitemia was similarly defined as *P. falciparum* asexual form detected on a thick film. After the inspection of 100 fields with no parasite found, the thick film was deemed negative. Placentas with malarial pigment in the absence of parasitemia were classified as negative for PM. Gravidity was divided into primigravid, secundigravid and multigravid (3 or more pregnancies). Newborn low-birth weight (<2500 g) was classified based on weight measured within 24 hours of birth. Preterm delivery was defined as occurrence at <37 weeks of gestation age assessed by the Ballard method [29]. Malaria transmission season comprised the months of April–June and October–December. The variable “malaria transmission season, third trimester” was defined as the women who encountered malaria transmission season during the last 3 months before delivery. The variable “anti-malarial use, third trimester” was based on the self-reported treatment during third trimester. Maternal anemia was defined as <11 g/dL and severe anemia as <7 g/dL at third trimester. CD4 cell counts were compared with cutoffs <350 cells/µl and <500 cells/µl. Maternal HIV-1 positive status was determined on the basis of antibodies to HIV-1, detected by two sequential rapid serological tests. Maternal viral-load was quantified and assigned values <1000 copies/ml, 1000–9999 copies/ml and ≥10,000 copies/ml.

#### Genetic definitions

For GM6, individual genotypes are GM6(+), GM6(−), and GM6(+/−), and for KM, they are KM1, KM3, and KM1-3 [18]. As previous studies have reported that GM and KM interactions, rather than GM or KM alone, play an important role in control of immune responses [20], [30], [31], [32], combinations of GM6 and KM genotypes were further used to explore the genetic association with PM in HIV-1 negative and positive women, respectively. The models for combination of genotypes in this study consist of the above three different individual GM6 genotypes combined with either (a) KM1-3(+) or (−), (b) KM1(+) or (−), or (c) KM3(+) or (−). Among these combination models, KM1-3(+) is KM1-3 heterozygote and KM1-3(−) includes KM1 and KM3 homozygotes. Using the model that includes two homozygous KM1 and KM3 in a single KM genotypic class (KM1-3(−)) enables us to examine the role of KM heterozygosity combined with GM6 genotypes in PM.

### Statistical Analysis

Because of the stratified enrollment in the main VT epidemiological study, all analyses were conducted in HIV-1 negative and HIV-1 positive women separately. Univariable analyses were based on Pearson χ^2^ tests for comparison of proportions and Wilcoxon rank sum tests were used for comparison of continuous distributions. Categorical and continuous variables included in the initial univariable analysis for characteristics were mother’s age, gravidity, maternal peripheral malaria, placental malaria pigment, preterm delivery, newborn birth weight, maternal anemia, anti-malarial use, malaria transmission season during third trimester, maternal CD4 count and maternal HIV-1 load. The effects of individual GM6 and KM genotypes, and combined genotypes on PM were analyzed using a multivariable logistic regression model controlling for gravidity, anti-malarial use, and malaria transmission season during the third trimester, all of which were significant in univariable analyses. GM6(−) and KM3 were used as references for the individual genotype analysis while GM6(−) with either KM1-3(−), KM1(−) or KM3(−) were used as references in the multivariable logistic regression analysis for the combined genotypes. Odds ratios (OR) with 95% confidence intervals (CI) were used to measure the strength of associations. All tests were two-tailed and statistical significance was defined as *P*<0.05. In addition, in order to evaluate the possible presence of selection, Chi-square goodness of fit tests for the departures from Hardy-Weinberg Equilibrium (HWE) in GM6 and KM genotypes and allele frequencies and the inbreeding coefficients (F(is)) were calculated based on the previously published formula [33], [34]. The HWE and F(is) were examined in whole study population and in four groups stratified by disease status. Data analysis was performed using SAS version 9.2 and the Hardy Weinberg package in R version 2.12.

## Results

### Characteristics of Study Participants

The profile of participants’ characteristics is summarized in Table 1 (n = 728). Since known and potential confounding variables associated with PM may differ by HIV-1 status, all the data analysis was conducted separately for HIV-1 negative and positive women. Our data showed that younger mothers were more likely to have PM than the older mothers (*P*<0.01), irrespective of their HIV-1 status. In HIV-1 negative women, univariable analysis showed that primigravid women were more likely to have PM than multigravid women. However, the gravidity-dependency of PM was not evident in HIV-1 positive women. Malaria transmission season during third trimester was found to be positively associated with PM. Irrespective of HIV-1 status, PM was statistically associated with maternal peripheral malaria presence during the third trimester and at delivery, malaria pigment present, and newborn birth weight <2500 g (Table 1). In HIV-1 positive women, PM was significantly associated with maternal anemia at delivery (<7 g/dL and <11 g/dL). Proportion of anti-malarial use during third trimester in HIV-1 positive women was higher in the women without PM than those with PM. Furthermore, HIV-1 positive women with PM were significantly more likely to have maternal HIV-1 viral-load copies/ml ≥10, 000 (Table 1).

**Table 1:** Characteristics of HIV-1 negative and HIV-1 positive women, by placental malaria (PM) status.

Category/Characteristics	HIV-1 negative women			HIV-1 positive women		
	With PM (n = 132)	Without PM (n = 107)	*P* a	With PM (n = 119)	Without PM (n = 370)	*P* a
Mother’s age, mean ± SD, years	19.5±3.7	22.3±4.6	**<0.01**	21.6±4.6	22.7±4.4	**<0.01**
Gravidity						
Primigravid	66.2	36.1	**<0.01**	43.7	33.2	0.11
Secundigravid	18.8	29.6	….	24.4	29.4	….
Multigravid	15.0	34.3	….	31.9	37.4	….
Maternal peripheral malaria						
Presence during third trimester	35.5	11.9	**<0.01**	52.4	11.9	**<0.01**
Presence at delivery	71.9	3.8	**<0.01**	67.8	2.5	**<0.01**
Malaria pigment present	88.0	1.9	**<0.01**	88.2	0.5	**<0.01**
Preterm delivery, <37 weeks	7.5	6.5	0.81	10.1	9.4	0.85
Newborn birth weight, <2500 g	6.8	0	**<0.01**	13.5	5.9	**0.01**
Maternal anemia at delivery, g/dL						
<7	7.0	3.8	0.39	13.4	6.3	**0.03**
<11	60.9	52.4	0.23	73.0	58.2	**<0.01**
Anti-malarial use, third trimester	22.7	30.8	0.18	19.5	33.9	**<0.01**
Place of living, semiurban (vs. urban)	33.8	23.6	0.09	31.4	25.7	0.24
Malaria transmission season, third trimester	74.4	61.1	**0.04**	57.1	51.9	0.34
Maternal CD4 count, cells/µl						
<350	….	….		18.9	18.8	0.99
<500	….	….		37.8	39.7	0.79
Median (interquartile range)	….	….		602 (422–841)	548 (376–798)	0.43
Maternal HIV-1 load, copies/ml						
<1000	….	….		41.2	49.3	**0.05**
1000–9999	….	….		30.6	34.7	….
≥10, 000	….	….		28.2	16.0	….
Median (interquartile range)	….	….		1884 (200–14,703)	1021 (200–4,740)	0.07

**Note:** Data are percentage of women, unless otherwise noted. PM, Placental malaria; SD, Standard deviation.

a *P* values for univariable analysis are based on either the Pearson χ*^2^* test for comparison of proportions, or the Wilcoxon rank sum test for comparison of continuous distributions.

### Analysis of the Effect of Individual GM6 and KM Genotypes on PM Infection with or without HIV-1

In univariable analysis, a borderline significant association between GM6(+) and GM6(+/−) genotypes and PM in HIV negative women was found, women without PM having higher frequencies of these genotypes (*P^a^* = 0.05, Table 2), but a similar association was not seen in HIV-1 positive women. There was no difference in the distribution of KM genotypes between women with PM and those without PM in both HIV-1 negative and HIV positive groups (Table 2). In multivariable analysis using GM6(−) and KM3 as references, the statistical significance earlier observed with GM6 genotypes disappeared after controlling for confounders (Table 3). Overall, there was no effect of the individual GM6 and KM genotypes on the risk of PM in either HIV-1 negative or positive women (Table 3).

**Table 2:** Distribution of individual GM6 and KM genotypes in HIV-1 negative and HIV-1 positive women, by PM status.

Category	HIV-1 negative women		HIV-1 positive women			
	With PM (n = 132)	Without PM (n = 107)	With PM (n = 119)	Without PM (n = 370)	*P* a	*P* b
GM6 genotype						
GM6(+)	10.5	19.6	5.1	10.4	**0.05**	0.18
GM6(+/−)	24.8	29.9	31.4	26.7	….	….
GM6(−)	64.7	50.5	63.5	62.9	….	….
KM genotype						
KM1	14.4	10.3	10.1	17.3	0.23	0.11
KM1-3	48.5	42.1	55.5	47.0	….	….
KM3	37.1	47.6	34.4	35.7	….	….

**Note:** Data are percentage of women. PM, Placental malaria.

a *P* value comparing PM+ and PM- in HIV-1 negative women.

b *P* value comparing PM+ and PM- in HIV-1 positive women.

**Table 3:** Effect of individual GM6 and KM genotypes on the risk for PM in HIV-1 negative and positive women.

Genotypes^a^	HIV-1 negative women			HIV-1 positive women		
	PM (%)^b^	Adjusted OR (95% CI)	*P* c	PM (%)^b^	Adjusted OR (95% CI)	*P* c
GM6(+)	40.0	0.47 (0.21 to 1.05)	0.065	13.6	0.47 (0.19 to 1.19)	0.11
GM6(+/−)	50.8	0.74 (0.39 to 1.41)	0.36	27.4	1.16 (0.73 to 1.86)	0.53
GM6(−)	61.4	1.00	….	24.5	1.00	….
KM1	63.3	1.48 (0.60 to 3.66)	0.39	10.1	0.62 (0.30 to 1.28)	0.19
KM1-3	58.7	1.52 (0.84 to 2.76)	0.16	27.5	1.25 (0.78 to 1.98)	0.35
KM3	49.0	1.00	….	23.7	1.00	….

**Note:** PM, Placental malaria; OR, odds ratios; CI, confidence interval.

a GM6(−) and KM3 are used as references respectively.

b For HIV negative women, one missing GM6 typing data, n = 238; for HIV positive women, five missing GM6 or KM typing data, n = 484.

c *P* values are derived from multivariate logistic regression, controlling for gravidity, anti-malarial use during third trimester, and malaria transmission season.

### Analysis of the Combined GM6 and KM Genotypes in Relation to Risk of PM with and without HIV-1

Since none of the GM6 and KM genotypes were by themselves significantly associated with PM in HIV-1 negative and positive women after adjusting for confounders, and since previous studies also showed an interactive effect of GM and KM in immune response [20], [30], [31], [32], we further analyzed 3 combinations of GM6 and KM genotypes (Tables 4 and 5). In HIV-1 negative women, there was no statistical association between PM and any combination of GM6 and KM1-3 (Table 4). Conversely, in HIV-1 positive women, the combination of GM6(+/−) and KM1-3(+) heterozygote was statistically significantly associated with increased risk of PM (OR, 2.08; 95% CI, 1.12–3.89) while the combination of GM6(+/−) and KM1-3(−) tended to be associated with decreased risk of PM (OR, 0.47; 95% CI, 0.22–1.00) (Table 4). Using the second combination (GM6 and KM3) in the analysis of HIV-1 negative women (Table 5), the combination of GM6(+) and KM3(+) homozygote was significantly associated with decreased risk of PM (OR, 0.25; 95% CI, 0.08–0.80) (Table 5). However, in HIV-1 positive women, the combination of GM6(+/−) and KM3(−) was associated with increased risk of PM (OR, 2.1; 95% CI, 1.18–3.73) (Table 5). This result in HIV positive women (Table 5) was partially in agreement with the finding in Table 4, suggesting that combined GM6(+/−) with either KM1-3 or KM1 render women more susceptible to PM in HIV-1 positive women. Analysis using the third combination of GM6 and KM1 genotypes showed no statistically significant association with PM (data not shown). In addition, any combination of GM6 and KM had no effect on other clinical outcomes such as placental malaria parasite density, maternal anemia <11 g/dl, low infant birth weight or gestational age in HIV-1 negative women (data not shown). In HIV-positive women, however, the combination of GM6(+/−) and KM3(+) was associated with increased risk for maternal anemia <7 g/dl (OR, 5.25; 95% CI, 1.30–21.2; *P* = 0.021) (data not shown).

**Table 4:** Effect of combined GM6 and KM1-3 genotypes on the risk for PM in HIV-1 negative and positive women.

Genotypes^a^	HIV-1 negative women			HIV-1 positive women		
	PM (%)	Adjusted OR (95% CI	*P* b	PM (%)	Adjusted OR (95% CI)	*P* b
GM6(+) KM1-3(+)	35.7	0.60 (0.18 to 2.05)	0.41	16.0	0.59 (0.19 to 1.87)	0.38
GM6(+) KM1-3(−)	42.9	0.62 (0.21 to 1.77)	0.37	10.5	0.29 (0.06 to 1.33)	0.11
GM6(+/−) KM1-3(+)	50.0	0.90 (0.36 to 2.28)	0.83	**42.2**	**2.08 (1.12 to 3.89)**	**0.021**
GM6(+/−) KM1-3(−)	52.9	1.04 (0.44 to 2.49)	0.93	**14.3**	**0.47 (0.22 to 1.00)**	**0.051**
GM6(−) KM1-3(+)	68.8	1.93 (0.92 to 4.07)	0.08	22.8	0.83 (0.47 to 1.41)	0.50
GM6(−) KM1-3(−)	54.7	1.00	….	26.1	1.00	….

**Note:** PM, Placental malaria; OR, odds ratios; CI, confidence interval.

a KM1-3(+) is KM1-3 heterozygote and KM1-3(−) includes KM1 and KM3 homozygotes. GM6(−)KM1-3(−) is used as reference.

b *P* values are derived from multivariable logistic regression, controlling for gravidity, anti-malarial use during third trimester, and malaria transmission season.

**Table 5:** Effect of combined GM6 and KM3 genotypes on the risk for PM in HIV-1 negative and positive women.

Genotypes^a^	HIV-1 negative women			HIV-1 positive women			
	PM (%)	Adjusted OR (95% CI)	*P* b	PM (%)	Adjusted OR (95% CI)		*P* b
**GM6(+) KM3(+)**	**35.3**	**0.25 (0.08 to 0.80)**	**0.019**	15.4	0.61 (0.13 to 2.89)	0.53	
GM6(+) KM3(−)	44.4	0.48 (0.16 to 1.44)	0.19	12.9	0.56 (0.18 to 1.71)	0.31	
GM6(+/−) KM3(+)	51.9	0.61 (0.24 to 1.57)	0.31	13.5	0.57 (0.24 to 1.36)	0.20	
GM6(+/−) KM3(−)	51.4	0.53 (0.23 to 1.26)	0.15	**36.6**	**2.10 (1.18 to 3.73)**	**0.011**	
GM6(−) KM3(+)	51.8	0.54 (0.26 to 1.15)	0.11	30.4	1.56 (0.91 to 2.71)	0.11	
GM6(−) KM3(−)	67.5	1.00	….	21.4	1.00	….	

**Note:** PM, Placental malaria; OR, odds ratios; CI, confidence interval.

a KM3(+) is KM3 homozygote while KM3(−) includes KM1 homozygote and KM1-3 heterozygote. GM6(−)KM3(−) is used as reference.

b *P* values are derived from multivariable logistic regression, controlling for gravidity, anti-malarial use during third trimester, and malaria transmission season.

### Hardy-Weinberg Equilibrium Tests

Malaria has a strong selective pressure on human genes [35], [36]. In this study, we observed that the combination of GM6(+) and KM3 homozygote was associated with decreased risk of PM in HIV-negative women while the combined heterozygosity of GM6(+/−) with either KM1-3 or KM1 was associated with increased risk of PM in HIV-positive women. In order to examine possible selection on GM6 and KM genes by diseases, departures from HWE and F(is) were measured in the Kenyan adult study population and further in the four disease groups (Tables 6 and 7). Overall, HWE tests show that there was no deviation for KM genotype frequency distribution in the whole study population (*P* = 0.75, F(is) = −0.01, n = 728) (Table 7) while there was significant deviation of GM6 genotype frequency distribution in the study population with positive F(is) (*P*<0.001, F(is) = 0.26, n = 725) (Table 6). When further stratified based on PM and HIV status for the HWE test, the trend remains similar: there was no deviation for KM genotype frequency distribution in the HIV-1 negative women regardless of PM status or in the HIV-1 positive women without PM except a borderline significance of deviation in HIV-1 positive women with PM showing negative F(is) (*P* = 0.05, F(is) = −0.18). In contrast, there were still significant deviations for GM6 genotype frequency distribution in the HIV-1 negative women with or without PM (*P*<0.001, F(is) = 0.30 and F(is) = 0.34 respectively) and in the HIV-1 positive women without PM (*P*<0.001, F(is) = 0.26), all of the three groups showing positive F(is) (Table 6). But interestingly, there was no significant deviation in HIV-1 positive women with PM for GM6 (*P* = 0.61, F(is) = 0.05).

**Table 6:** Hardy-Weinberg Equilibrium Test for GM6 genotypes.

GM6 Group	Observed Genotypes (n)				Allele Frequency		Expected Genotypes (n)			Hardy Weinberg Results		Inbreeding Coefficient
	GM6 −	GM6+/−	GM6+	Total (n)	GM6 −	GM6+	GM6 −	GM6+/−	GM6+	χ^2^	P value	F(is)
All women	446	200	79	725	0.75	0.25	411.2	269.6	44.2	48.3	<0.001	0.26
HIV+/PM+	75	37	6	118	0.79	0.21	74.1	38.8	5.1	0.3	0.61	0.05
HIV+/PM−	231	98	38	367	0.76	0.24	213.6	132.8	20.6	25.2	<0.001	0.26
HIV−/PM+	86	33	14	133	0.77	0.23	79.0	47.0	7.0	11.8	<0.001	0.30
HIV−/PM−	54	32	21	107	0.65	0.35	45.8	48.4	12.8	12.3	<0.001	0.34

Chi-square tests were used to determine deviation from HWE, and F(is) was calculated based on the formula F = 1– Het/Het_HW_, where Het is the number of heterozygotes observed, and Het_HW_ is the number of heterozygotes expected under HWE [33], [34].

**Table 7:** Hardy-Weinberg Equilibrium Test for KM genotypes.

KM Group	Observed Genotypes (n)				Allele Frequency		Expected Genotypes (n)			Hardy Weinberg Results		Inbreeding Coefficient
	KM 1	KM 1,3	KM 3	Total (n)	KM 1	KM 3	KM 1	KM 1,3	KM 3	χ^2^	P value	F(is)
All women	106	349	273	728	0.39	0.61	108.1	344.8	275.1	0.1	0.75	−0.01
HIV+/PM+	12	66	41	119	0.38	0.62	17.0	56.0	46.0	3.8	0.050	−0.18
HIV+/PM−	64	174	132	370	0.41	0.59	61.6	178.8	129.6	0.3	0.61	0.03
HIV−/PM+	19	64	49	132	0.39	0.61	19.7	62.6	49.7	0.1	0.80	−0.02
HIV−/PM−	11	45	51	107	0.31	0.69	10.5	46.0	50.5	0.1	0.82	0.02

Chi-square tests were used to determine deviation from HWE, and F(is) was calculated based on the formula F = 1– Het/Het_HW_, where Het is the number of heterozygotes observed, and Het_HW_ is the number of heterozygotes expected under HWE [33], [34].

## Discussion

In this study, we report an association of GM6 and KM genotypes on the risk of PM in HIV-1 infected and uninfected pregnant women. Although none of the individual GM6 and KM genotypes were by themselves significantly associated with PM, the combination of homozygous GM6(+) and KM3 was significantly associated with decreased risk of PM infection in HIV negative women. In HIV-1 positive women, the combination of heterozygous GM6(+/−) with either KM1-3 or KM1 revealed an increased risk of PM. These results suggest that GM6 and KM genotypes, two unlinked genetic loci, could interact to influence the susceptibility to PM infection in HIV-1 negative and positive women by regulation of disease-specific humoral responses.

The combination of GM6(+) and KM3 homozygotes may possibly affect susceptibility to PM infection in HIV-1 negative women through immune regulation to influence the specificity, subclass switch and titer of anti-malarial antibody responses. GM markers are located on the constant region and there is evidence for the involvement of these regions in antibody specificity with the variable region, probably through the formation of idiotypic determinants, modulation of antibody binding affinity and linkage disequilibrium with the variable epitopes [28], [37]. A previous study conducted in non-pregnant Sudanese (all ages) showed that the carriers of GM1,17 (present on IgG1); 5,13,14,6 (present on IgG3) phenotype were associated with higher incidence of malaria and higher baseline levels of total IgG and non-cytophilic IgG subclasses than the non-carriers [38]. This same study further suggested that the above-mentioned implication of GM 1,17; 5,13,14,6 phenotype might be mainly due to the GM6 allotype involvement. Although there was an indication of limited involvement of KM allotype in susceptibility to malaria infection and antibody responses in this previous study [38], the current study showed that the combination GM6(+) and KM3(+) homozygote plays a role in the decreased risks of PM infection in HIV negative women. This could be due to the difference in the study models used, pregnant women vs. general population. In addition, a more recent study in Beninese children showed an age-related impact of GM 5,6,13,14;1,17 or KM1 phenotype on malaria specific cytophilic IgG responses [39]. The interactive effect of GM and KM phenotypes in the clearance and persistence of hepatitis C infection has also been reported [19], [20], suggesting that Ig GM and KM allotypes are important immuno-genetic factors in infectious diseases. Functionally, the γ and κ chains carrying specific GM and KM allotypes by nonrandom pairing could form a paratope with the necessary structure for recognition [30] of the malaria antigen epitopes. Importantly, GM allotypes may further interact with Fcγ receptors (FcγR) to affect disease risk or protection. All GM epitopes including GM6, with the exception of G1M3 and 17, are found on the Fc-portion of the IgG molecule [20]. It is possible that particular FcγR and GM6 alleles epistatically interact [17] to influence the susceptibility to PM infection. Our previous study showed that FcγRIIa-H/H131 receptor for immunoglobulin G is associated with susceptibility to PM [15]. An *in vitro* study conducted by others reported that pregnancy-associated VSA-specific cytophilic IgGs promote phagocytic clearance of parasite infected erythrocytes, suggesting the contribution of this immune effector mechanism in the decrease of parasite load in the placenta [40]. It is known that IgG opsonized phagocytosis of *P. falciparum* infected erythrocytes could be mediated by FcγRI and FcγRIIa [41]. In addition, the potential effect of the cytophlic IgG3 involving Fcγ receptors in protection against *P. falciparum* malaria through phagocytosis of merozoites, neutrophil respiratory bursts and monocyte-mediated growth inhibition have been established in naturally acquired immunity [42], [43], [44].

In HIV-1 positive women the combination of heterozygous GM6(+/−) with either KM1-3 or KM1 was associated with susceptibility to PM infection. This suggests that HIV-1 infection could influence the relationship between GM and KM allotypes and PM infection, possibly by altering malaria-specific antibody responses. Indeed, several studies in pregnant women have reported that HIV infection decreases frequency and concentration of malaria-specific antibodies and/or changes balance of IgG subclasses against various antigens including pre-erythrocytic and blood stage antigens as well as pregnancy-associated VSA with impairment of opsonic phagocytic clearance of parasites [13], [14], [40]. This could lead to the increased risk of PM infection in HIV-1 positive women. In this study, we also observed that the combination of heterozygous GM6(+/−) again with different KM, homozygous KM3(+), was associated with maternal anemia (Hb<7 g/dL) in HIV-1 positive women. Although the actual mechanism by which the combination of GM6(+/−) and KM3 would regulate anemia in pregnant women is not clear, the result indicates that GM6(+/−) could play a role in the risk of severe maternal anemia, one symptom of malaria during pregnancy.

We also measured departure from HWE in GM6 and KM allele frequencies and F(is) in the study population to evaluate possible selection [33], [45], [46] by malaria on GM6 and KM as malaria has a strong selection on human genes [35], [36]. HWE tests clearly showed that there was no deviation for KM genotype frequency distribution in the study population. In contrast, an overall significant deviation of GM6 genotype frequency distribution was observed in the same population, showing significant positive F(is). Positive F(is) indicates a deficit on heterozygotes in the population [34]. Most importantly, when further stratified by PM and HIV status there were still significant positive F(is) for GM6 in the HIV-1 negative women regardless of PM and in the HIV-1 positive women without PM while there was no significant deviation from HWE in HIV-1 positive women with PM. Tests of departure from HWE and F(is) have been used for different purposes; some have used the tests for searching disease-susceptibility gene loci and detecting selection, others have used them for genotyping quality control [33], [45], [46], [47]. However, HWE and F(is) results from our study with distinct patterns between GM6 and KM and with different strength among disease groups for GM6 were unlikely due to genotype errors as we employed different quality control measures for GM6 genotyping (see method section). In addition, our study was not a typical case-control study; rather, a diseases-stratified enrollment was used (see method section). Most likely, GM6 showing overall significant positive F(is) indicates low heterozygote fitness in the population possibly shaped by selection over time [33], [34]. The results of no significant departure from HWE in GM6 genotype frequency in HIV-1 positive women with PM but strong positive F(is) in other three groups further suggest that the group without low heterozygote fitness has less potential for resistance to malaria and/or HIV-1 infection. This notion could be partially explained by the results from our association analysis above showing GM6(+) involvement in the association with decreased risk of PM (homozygote advantage) while GM6(+/−) was associated with increased risk of PM (heterozygote disadvantage) regardless of HIV-1 status. As such, GM6 has fitness value likely selected by malaria infection. The assertion that GM6 is under selection by malaria is also supported by the fact that the geographic distribution of GM6 related haplotypes coincides with the region of high falciparum malaria and sickle-cell allele prevalence [24].

Taken together, this study highlights the association between GM6 and KM genotypes with PM in HIV-1 negative and HIV-1 positive women. These findings suggest that the combination of homozygous GM6(+) and KM3 may protect against PM in HIV-1 negative women, while the HIV-1 positive women with heterozygous GM6(+/−) combined with KM1-3 or KM1 may be more susceptible to PM. In addition, the result of a deficit in heterozygotes for GM6 in our study population (Kenyan pregnant women) suggests that low GM6 heterozygous fitness could be due to selection mainly by malaria infection. However, more studies in other populations and in different malaria endemic areas are needed to validate this finding.

### Presentation of Results

This result was presented in part at the 59th Annual ASTMH meeting, November 3–7, 2010, in Atlanta, Georgia, USA.
